# Prolonged Intestinal Ethanol Absorption and Oxidative Stress: Revisiting the Gut–Liver Axis in Alcohol-Associated Disease

**DOI:** 10.3390/ijms26125442

**Published:** 2025-06-06

**Authors:** Beom Sun Chung, Keungmo Yang, Chihyun Park, Tom Ryu

**Affiliations:** 1 Department of Anatomy, Yonsei University Wonju College of Medicine, Wonju 26426, Republic of Korea; bschung@yonsei.ac.kr; 2 Department of Internal Medicine, Division of Gastroenterology and Hepatology, College of Medicine, The Catholic University of Korea, Seoul 06591, Republic of Korea; yang27jin@catholic.ac.kr; 3 Forensic Chemistry Division, National Forensic Service, Wonju 26460, Republic of Korea; pch0938@korea.kr; 4 Department of Internal Medicine, Institute for Digestive Research, Digestive Disease Center, Soonchunhyang University College of Medicine, Seoul 04401, Republic of Korea

**Keywords:** redox imbalance, alcohol metabolism, gut–liver axis, oxidative stress, cytochrome P450 2E1, microsomal ethanol oxidizing system, intestinal permeability

## Abstract

Chronic alcohol consumption induces oxidative stress not only in the liver but also in the gastrointestinal tract, where prolonged intestinal ethanol absorption plays a pivotal and underrecognized role. This review reframes ethanol pharmacokinetics to emphasize sustained jejunal and ileal uptake, which maintains elevated blood alcohol levels and perpetuates redox imbalance across the gut–liver axis. We integrate recent findings on ethanol-induced barrier dysfunction, CYP2E1-mediated ROS production, microbial dysbiosis, and mitochondrial disruption, proposing that the intestine is an active site of injury and a driver of systemic inflammation. Key mechanistic insights reveal that gut-derived endotoxins, compromised epithelial integrity, and microbiome–mitochondria interactions converge to exacerbate hepatic and extrahepatic damage. We further explore emerging therapeutic strategies—ranging from NAD^+^ repletion and probiotics to fecal microbiota transplantation—that target this upstream pathology. Recognizing prolonged intestinal ethanol absorption as a clinically meaningful phase offers new directions for early intervention and redox-based treatment in alcohol-associated disease.

## 1. Introduction

Alcohol consumption, whether occasional or habitual, has long been known to disrupt redox balance across various tissues [1]. The liver, due to its central role in ethanol metabolism, has traditionally been regarded as the primary site of alcohol-induced oxidative stress and injury. As a result, most models of alcohol-related disease have focused on hepatic metabolism as the main determinant of systemic toxicity. However, recent research has increasingly highlighted the gastrointestinal (GI) tract, especially the small intestine, as a metabolically active and vulnerable site during ethanol exposure [2].

A less commonly emphasized feature of ethanol pharmacokinetics is its prolonged absorption within the small intestine, particularly under conditions of delayed gastric emptying [3]. This mechanism prolongs systemic exposure beyond the initial drinking episode and may play a critical role in shaping redox homeostasis. Although the term “intestinal drinking” has been proposed to describe this sustained intestinal phase, we use it here primarily as a conceptual tool to contextualize a range of recent findings [4,5]. Indeed, this phase of prolonged intestinal ethanol absorption has implications for alcohol clearance kinetics, tissue-specific oxidative damage, and ethanol–drug interactions. Its significance lies not only in metabolic extension but in the temporal decoupling between drinking behavior and peak systemic effects—a gap with profound clinical relevance.

Classic models emphasize the enzymatic conversion of ethanol by alcohol dehydrogenase (ADH) and aldehyde dehydrogenase (ALDH), resulting in the production of acetaldehyde, acetate, and reactive oxygen species (ROS) [6,7]. These reactions disturb the NAD^+^/NADH ratio and promote hepatic inflammation. However, they do not fully capture the contribution of the intestinal epithelium, where CYP2E1, part of the microsomal ethanol oxidizing system (MEOS), is also induced by ethanol. This enzyme complex generates ROS locally and contributes to oxidative stress in the gut. In this context, the gut becomes not only a site of absorption but also a critical organ of metabolic amplification and redox dysregulation. The presence of NADPH-cytochrome P450 reductase and phospholipid-rich endoplasmic reticulum membranes in enterocytes further intensifies ROS production.

In rodent models using intragastric ethanol infusion, ethanol retention in the intestine has been shown to occur especially under high-fat diets or pharmacologic modulation of motility [8,9]. These studies report a prolonged elevation in blood alcohol concentration (BAC), implicating the small intestine as a major site of delayed systemic ethanol absorption. The consequence is a prolonged oxidative burden on both intestinal and hepatic tissues [10]. This shift in absorption site alters first-pass metabolism dynamics and extends systemic exposure, compounding redox stress and metabolic demand in extrahepatic tissues.

In enterocytes, ROS generated via CYP2E1 and MEOS impair mitochondrial function and damage proteins involved in maintaining barrier integrity, including tight junction components such as occludins and claudins [11,12]. This loss of barrier function facilitates the translocation of lipopolysaccharide (LPS) and other microbial products into the portal circulation, where they activate hepatic macrophages (Kupffer cells) and provoke inflammatory responses [13]. Increased intestinal permeability also augments vulnerability to microbial dysbiosis, enhancing the survival and expansion of LPS-rich taxa.

Chronic ethanol intake profoundly alters the composition of the gut microbiota, leading to a marked shift that favors the overgrowth of LPS-producing Gram-negative bacteria while concurrently depleting beneficial anaerobic species essential for maintaining intestinal barrier integrity [14,15]. This dysbiotic environment is of considerable importance because it sets the stage for a heightened pro-inflammatory milieu within the gut lumen, significantly escalating the oxidative damage inflicted upon the intestinal epithelium. Such alterations are not merely localized disruptions; they constitute a critical driver of systemic pathophysiology by creating a vicious feedback loop in which ongoing intestinal dysfunction exacerbates hepatic injury. Recent advances in the field have further underscored the pivotal role of microbial metabolites in modulating mitochondrial biogenesis and detoxification pathways within host tissues, suggesting that the gut microbiome’s influence extends beyond the gut to exert a regulatory impact on overall cellular homeostasis. The recognition of this microbiota-driven signaling underscores the gut–liver axis as a dynamic interface that can either uphold redox balance or, when disturbed, propagate injury throughout the entire organism.

Although this prolonged intestinal absorption phase is not widely considered in clinical assessment, it has implications for interpreting BAC curves, assessing hangover severity, and understanding delayed drug–alcohol interactions [16,17]. It also provides insight into how alcohol consumption alters the kinetics and effects of concurrently ingested xenobiotics. The metabolic consequences of prolonged intestinal ethanol absorption may be further shaped by co-exposures to dietary fat, xenobiotics, or microbial metabolites, making this a complex but highly relevant frontier. This phase may also contribute to individual variability in alcohol tolerance and toxicity profiles.

We propose that the small intestine should be regarded not as a passive conduit but as a key contributor to alcohol-associated systemic oxidative stress [18]. Interventions aimed at preserving intestinal barrier function, modulating microbial composition, or accelerating ethanol clearance from the gut may provide opportunities for preventing or mitigating ethanol-related tissue injury. These strategies are investigational, but growing evidence suggests they could complement conventional antioxidant and abstinence-based therapies [19,20]. In particular, therapies that target intestinal redox signaling and epithelial–microbial crosstalk may interrupt the gut–liver injury loop before hepatic dysfunction ensues.

**Figure 1:**
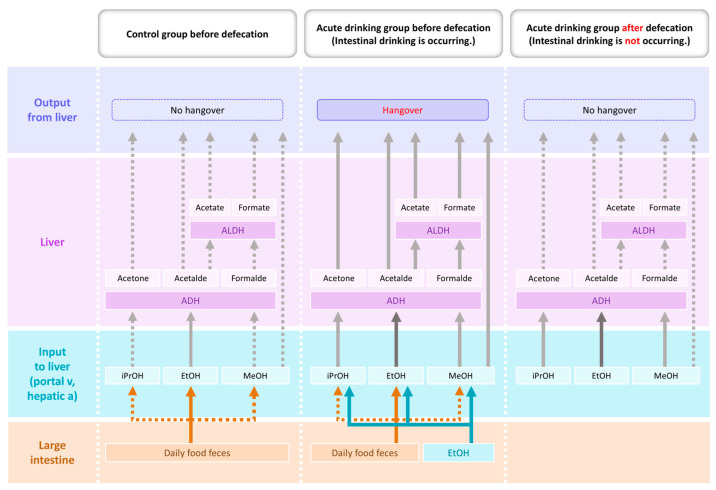
Schematic diagram showing the proposed model depicting effect of defecation on hangover the day after binge ethanol drinking. Ethanol absorption from the large intestine ceases after defecation, thereby halting acetaldehyde production and enabling the degradation of hangover-related compounds, such as acetaldehyde, methanol, and isopropanol. Abbreviations: EtOH, ethanol; MeOH, methanol; iPrOH, isopropanol; Acetalde, acetaldehyde; Formalde, formaldehyde; ADH, alcohol dehydrogenase; ALDH, aldehyde dehydrogenase.

This review aims to bridge gaps among recent findings in ethanol pharmacokinetics, redox biology, and gut–liver axis research. By synthesizing these insights, we outline a framework in which sustained and prolonged intestinal ethanol absorption functions as a critical driver of redox imbalance and systemic inflammation in alcohol-associated disease (Figure 1). This systems-level approach may yield novel targets for early intervention and enhance our mechanistic understanding of gut-centered pathogenesis in alcohol use disorders.

## 2. Ethanol Metabolism and Redox Imbalance

Ethanol absorption is classically associated with the stomach and proximal small intestine, where rapid uptake occurs under fasting conditions. However, under typical dietary and postprandial states, gastric emptying is slowed, and a significant portion of ethanol reaches the jejunum and ileum [21,22]. This transition shifts the site of absorption distally, resulting in a slower, more sustained ethanol uptake profile. Such kinetic alterations influence the peak blood alcohol concentration (BAC), duration of exposure, and the extent of metabolic disruption [23,24]. These changes are especially important in understanding systemic toxicity, as ethanol’s absorption kinetics influence downstream tissue exposure and metabolic overload.

In both animal models and human studies, delayed gastric emptying—induced by high-fat meals, opioid medications, or diabetes—prolongs the transit time of ethanol into the small intestine. In these contexts, ethanol continues to be absorbed over several hours, maintaining elevated BAC levels despite cessation of drinking [25,26]. This extended exposure phase corresponds to sustained oxidative activity in both intestinal and hepatic tissues, contributing to systemic redox imbalance [27]. Repeated exposure under these conditions may lead to cumulative mitochondrial dysfunction and lipid peroxidation.

Moreover, ethanol absorption from the small intestine is facilitated by its high lipid solubility and passive diffusion across the enterocyte membrane. Once absorbed, ethanol can directly engage intestinal enzymes, including CYP2E1, initiating local oxidative stress [28]. These metabolic reactions occur concurrently with hepatic processing and are especially pronounced in individuals with compromised gastric motility. The interplay between gastrointestinal transit, mucosal enzyme expression, and ethanol burden determines the intensity and duration of oxidative damage.

Recent pharmacokinetic studies have shown that the duration and magnitude of systemic ethanol exposure can vary dramatically depending on intestinal transit time and individual differences in CYP2E1 activity. Mathematical modeling of BAC profiles reveals a biphasic absorption pattern when ethanol persists in the small intestine, challenging conventional views that attribute ethanol clearance solely to hepatic metabolism [29,30,31]. This understanding calls for a shift in evaluating ethanol kinetics not just in terms of peak levels but also in terms of prolonged systemic engagement.

Although the term “intestinal drinking” remains conceptual, it reflects a reproducible physiological pattern with implications for alcohol pharmacokinetics and toxicity. Recognizing this phase expands our understanding of ethanol’s systemic effects, particularly in vulnerable populations, such as individuals with metabolic syndrome, alcohol use disorder, or chronic gastrointestinal conditions [32,33]. Prolonged intestinal retention also raises concern for synergistic toxicity with substances that share metabolic pathways.

Furthermore, sustained intestinal ethanol absorption may affect the pharmacokinetics of co-ingested substances metabolized through shared pathways, including CYP2E1 and ALDH, heightening the risk of drug–alcohol interactions [34,35]. This includes both pharmaceutical agents and environmental toxins that depend on redox-sensitive metabolic enzymes.

Finally, systemic oxidative stress originating from the intestine can disrupt redox-sensitive pathways in remote organs and prolong the inflammatory cascade well beyond ethanol clearance. This includes altered mitochondrial dynamics, impaired barrier regeneration, and modulation of innate immune signaling [36,37,38,39,40]. The oxidative burden is thus not only local but systemic, establishing the gut as a persistent contributor to alcohol-related multi-organ injury.

By characterizing ethanol pharmacokinetics beyond the stomach, we highlight a critical but underappreciated determinant of redox balance and tissue injury. This section lays the foundation for understanding how sustained ethanol absorption from the intestine propagates oxidative stress along the gut–liver axis, requiring an integrative metabolic and immunologic response framework.

## 3. Prolonged Intestinal Ethanol Absorption and Redox Imbalance

Prolonged exposure to ethanol within the small intestine leads to a distinct set of localized injuries, primarily driven by redox imbalance. The metabolic activity of enterocytes plays a key role in this context, especially given their capacity to express ethanol-metabolizing enzymes, such as CYP2E1. Unlike hepatic cells, intestinal epithelial cells are in direct contact with luminal ethanol and microbial products, making them uniquely vulnerable to oxidative insults [41,42]. Their constant exposure to fluctuating redox conditions, microbial metabolites, and dietary components renders the intestinal epithelium a site of dynamic metabolic stress.

Ethanol metabolism by CYP2E1 in enterocytes generates significant quantities of reactive oxygen species (ROS), including superoxide and hydrogen peroxide. These ROS are produced in proximity to mitochondrial membranes, where they compromise membrane potential, disrupt ATP synthesis, and trigger mitochondrial permeability transition (MPT) [43]. Such disruptions promote cytochrome c release and caspase activation, initiating apoptosis in the gut epithelium [44]. These effects are exacerbated by ethanol-induced mitochondrial fragmentation and oxidative phosphorylation impairment.

Importantly, chronic alcohol exposure has been shown to downregulate aldehyde dehydrogenase 2 (ALDH2) in intestinal tissues, reducing the capacity to detoxify acetaldehyde, a highly reactive intermediate [25]. Elevated acetaldehyde levels exacerbate oxidative damage by forming protein adducts and impairing redox-sensitive transcription factors, such as Nrf2, which regulates antioxidant gene expression [45,46]. The inhibition of Nrf2-mediated signaling results in reduced expression of glutathione peroxidase and heme oxygenase-1, further weakening the antioxidant defense system.

In addition to epithelial apoptosis, oxidative stress alters autophagic flux and disrupts tight junction dynamics. Proteins such as ZO-1, claudin-1, and occludin undergo oxidative modifications that impair their structural stability, leading to increased intestinal permeability [47]. This disruption allows luminal toxins and microbial fragments to bypass mucosal defenses and enter systemic circulation. Chronic ethanol exposure may also inhibit epithelial cell turnover and regeneration, compounding barrier deterioration.

The translocated LPS acts as a potent stimulator of Toll-like receptor 4 (TLR4) on hepatic Kupffer cells, initiating an inflammatory cascade that propagates liver injury [48]. Simultaneously, LPS and other endotoxins activate pattern recognition receptors (PRRs) in enteric immune cells, promoting the release of pro-inflammatory cytokines, such as IL-6, IL-1β, and TNF-α [49]. These inflammatory mediators further compromise barrier function. They enhance oxidative injury and create a self-reinforcing loop of damage. Enteric glial activation and mucosal neuroimmune modulation may also contribute to persistent inflammatory signaling.

Another consequence of oxidative stress in the gut is lipid peroxidation. Polyunsaturated fatty acids within epithelial cell membranes react with ROS to form malondialdehyde (MDA) and 4-hydroxynonenal (4-HNE), which can crosslink proteins and DNA, amplifying cellular dysfunction [50]. Mitochondria themselves are particularly susceptible to this lipid peroxidation, leading to progressive energy failure in the gut mucosa [51]. Lipid peroxidation products may also function as secondary messengers, further perpetuating oxidative signaling.

The role of autophagy in intestinal redox regulation is complex but increasingly recognized. Under physiological conditions, autophagy helps maintain epithelial integrity by removing damaged mitochondria and oxidized proteins. However, chronic ethanol exposure impairs this protective mechanism, shifting the balance toward cell death and inflammation [52]. Recent studies suggest that disrupted mitophagy may contribute to persistent ROS leakage and immune cell activation.

The net effect of these processes is a leaky, inflamed intestinal barrier that acts as a persistent source of systemic oxidative stress. This condition not only contributes to hepatic injury but also primes distant organs, such as the brain and pancreas, for secondary injury through circulating endotoxins and cytokines [53,54]. Additionally, intestinal inflammation can alter systemic glucose and lipid metabolism, exacerbating comorbid metabolic conditions.

Together, these findings illustrate how the intestine transforms from a site of ethanol absorption into a generator of redox imbalance and systemic pathology. Understanding these localized injuries is essential for developing interventions aimed at restoring gut integrity and interrupting the gut–liver inflammatory circuit [55,56,57]. Sustained intestinal ethanol absorption thus represents a pathophysiological amplifier that intensifies alcohol-related disease severity and therapeutic resistance.

## 4. Gut–Liver Axis and Redox Amplification

Once microbial byproducts and inflammatory signals breach the intestinal barrier, they are rapidly conveyed to the liver via the portal vein. This anatomical arrangement places the liver in direct and continuous contact with gut-derived insults, particularly lipopolysaccharide (LPS), peptidoglycans, and bacterial DNA. These pathogen-associated molecular patterns (PAMPs) engage hepatic pattern recognition receptors (PRRs), including Toll-like receptors (TLRs) and nucleotide-binding oligomerization domain-like receptors (NLRs), thereby initiating potent inflammatory cascades [58]. The magnitude of this immune signaling depends on hepatic microenvironmental factors, such as oxygen gradients, sinusoid architecture, and redox tone.

Among these pathways, activation of the TLR4–NF-κB axis in Kupffer cells is a central event in alcohol-associated liver disease (ALD). TLR4 stimulation by LPS induces transcription of TNF-α, IL-1β, and IL-6, leading to local hepatocellular injury, recruitment of monocyte-derived macrophages, and promotion of steatohepatitis [59]. Furthermore, chronic ethanol exposure primes hepatic immune cells for exaggerated responses to PAMPs by downregulating suppressor molecules, such as IRAK-M and SOCS1, compounding the inflammatory milieu [60]. This phenomenon of immune sensitization reflects a broader failure in hepatic immunotolerance.

Concomitant with immune activation, hepatic redox balance is disrupted through several interconnected mechanisms. Following prolonged alcohol consumption, mitochondrial acetaldehyde oxidation is diminished, impairing the organelle’s capacity to metabolize ethanol. Oxidative stress and the formation of acetaldehyde-protein adducts further compromise mitochondrial β-oxidation. In parallel, the induction of CYP2E1 in hepatocytes sustains reactive oxygen species (ROS) production and lipid peroxidation [61]. This cumulative mitochondrial dysfunction precipitates ATP depletion, exacerbates endoplasmic reticulum (ER) stress, and heightens cellular susceptibility to pro-apoptotic signals. Additionally, glutathione stores—essential for neutralizing ROS—are significantly depleted during chronic ethanol exposure, further destabilizing redox homeostasis.

Recent studies have highlighted the role of bile acid signaling in coordinating gut–liver crosstalk. Ethanol alters the expression and function of nuclear receptors, such as farnesoid X receptor (FXR) and Takeda G-protein receptor 5 (TGR5), which regulate bile acid synthesis, microbial composition, and epithelial integrity [62]. FXR suppression impairs the secretion of antimicrobial peptides and disrupts enterohepatic circulation, enhancing microbial overgrowth and translocation. Additionally, TGR5 downregulation may impair adaptive thermogenesis and intestinal immune regulation.

Hepatic stellate cells (HSCs), the main fibrogenic cells in the liver, are also key responders to this toxic axis. They become activated in response to LPS, ROS, and inflammatory cytokines, secreting extracellular matrix proteins that drive fibrosis. This transition from quiescent to myofibroblast-like phenotypes is regulated by TGF-β1 signaling, mitochondrial ROS, and inflammasome activation [63]. Progressive activation of HSCs is a hallmark of chronic liver disease and a major contributor to cirrhosis development.

Of note, redox imbalance does not remain confined to the liver but extends systemically. Circulating levels of oxidized glutathione, lipid peroxides, and protein carbonyls are elevated in patients with alcohol use disorder, reflecting widespread oxidative injury [64]. This systemic redox disruption contributes to neuroinflammation, cardiovascular risk, and immune dysregulation in alcohol-related pathology. Immune-metabolic coupling is altered, with increased leukocyte ROS production and impaired regulatory T-cell responses.

Transcriptional profiling and redox proteomics have revealed altered expressions of antioxidant enzymes and mitochondrial respiratory proteins in both hepatic and extrahepatic tissues. These findings suggest that therapeutic approaches should aim not only to suppress inflammation but also to restore redox equilibrium at multiple organ levels [65,66]. Nutritional support targeting redox co-factors, such as NAD^+^ and glutathione precursors, may also enhance therapeutic response.

**Figure 2:**
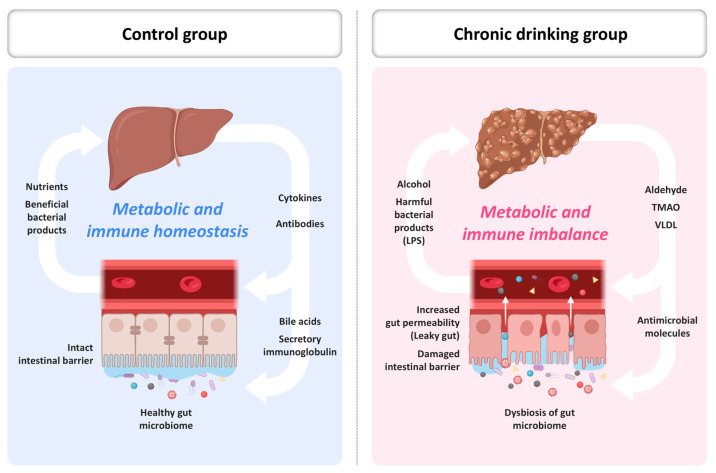
Schematic diagram of the gut–liver axis and dysbiosis resulting from chronic alcohol consumption. LPS, lipopolysaccharide; TMAO, trimethylamine-N-oxide; VLDL, very low-density lipoprotein.

This gut–liver signaling circuit encompasses barrier dysfunction, microbial translocation, immune activation, bile acid dysregulation, and systemic oxidative amplification [14,57,67]. The circuit represents a convergence point where microbial, metabolic, and inflammatory signals coalesce to drive liver injury and systemic disease. In sum, the gut–liver axis functions as a conduit for both metabolic integration and inflammatory escalation in the context of ethanol exposure. Understanding its molecular pathways opens opportunities for targeted interventions aimed at preserving organ integrity and mitigating systemic disease progression (Figure 2).

## 5. Possible Therapeutic Strategies Targeting the Gut–Liver Redox Axis

Ethanol-induced oxidative stress in the intestinal epithelium not only impairs local barrier function but also triggers complex immunometabolic adaptations. These responses are mediated by epithelial cells, immune cells, and microbial signals acting in concert to shape local and systemic immunity. One of the earliest consequences of oxidative injury in enterocytes is the activation of redox-sensitive transcription factors, such as NF-κB and AP-1, which stimulate the production of chemokines and cytokines that recruit immune cells to the intestinal mucosa [68]. This molecular signaling initiates the first phase of mucosal immune infiltration and primes a broader inflammatory environment.

Neutrophils and macrophages are the first responders to ethanol-induced mucosal damage. Upon activation, they release additional ROS, nitrogen species, and matrix metalloproteinases (MMPs), exacerbating tissue injury and prolonging inflammation. These innate responses are further amplified by the recruitment and activation of dendritic cells (DCs) and monocyte-derived macrophages, which function as antigen-presenting cells and secrete pro-inflammatory mediators, such as TNF-α and IL-23 [69]. These events not only damage epithelial architecture but also disrupt microbiota–immune equilibrium, worsening local redox imbalance.

This inflammatory environment profoundly influences adaptive immunity. Ethanol exposure alters the balance between regulatory T cells (Tregs) and pro-inflammatory Th17 cells. A decrease in IL-10-producing Tregs and expansion of IL-17-secreting Th17 cells within the lamina propria have been reported, particularly in chronic alcohol exposure models. This shift skews mucosal immunity toward a pro-inflammatory phenotype, facilitating barrier disruption and microbial translocation [70]. Th17-mediated inflammation also contributes to extraintestinal manifestations, including autoimmune exacerbation and liver fibrogenesis.

Oxidative stress also impairs immunoglobulin A (IgA) production by intestinal plasma cells, weakening mucosal defense and allowing luminal pathogens and metabolites to more easily access the epithelium. IgA deficiency has been associated with greater microbial diversity, increased endotoxin load, and more severe liver pathology in preclinical models of alcoholic liver disease [71]. The depletion of secretory IgA may also destabilize microbial biofilm architecture, encouraging pathogen overgrowth and LPS release.

The metabolic state of immune cells, termed immunometabolism, is increasingly recognized as a key determinant of their function in alcohol-related disease. Ethanol and its metabolites modulate pathways, such as glycolysis, fatty acid oxidation, and mitochondrial respiration within immune cells, influencing their phenotype and cytokine profiles. For example, pro-inflammatory macrophages (M1-like) exhibit increased glycolysis and ROS production, whereas anti-inflammatory macrophages (M2-like) rely on oxidative phosphorylation and lipid metabolism [72]. Ethanol skews this balance in favor of M1 polarization, perpetuating a pro-inflammatory niche that destabilizes gut homeostasis.

Moreover, systemic exposure to microbial metabolites, such as LPS and peptidoglycans, activates inflammasomes in liver-resident macrophages and circulating monocytes. This leads to the release of IL-1β and IL-18, which further amplify hepatic and intestinal inflammation. Inflammasome activation is driven by both mitochondrial ROS and cytosolic NAD^+^ depletion, linking redox imbalance to immune dysfunction [73]. These responses are interconnected with intestinal cell death pathways, autophagy impairment, and metabolic reprogramming.

Importantly, several immunomodulatory interventions have shown promise in mitigating these responses. NAD^+^ precursors, such as nicotinamide riboside (NR) and NMN, have been shown to restore redox balance and suppress inflammatory signaling by enhancing sirtuin activity and promoting mitochondrial health. Similarly, emerging evidence suggests that administration of FXR agonists and probiotics can attenuate ethanol-induced mucosal inflammation by restoring microbial balance and bile acid signaling [74,75]. Dual-acting formulations that combine bile acid analogs with prebiotic fibers are also under investigation.

Emerging data support the role of dietary polyphenols, such as resveratrol, quercetin, and curcumin, in modulating intestinal immunity. These compounds activate Nrf2 signaling, inhibit NF-κB, and enhance tight junction integrity, offering a multifaceted approach to redox and immune restoration. Polyphenol metabolites generated by gut bacteria may exert localized effects at the epithelial interface. Combined strategies that integrate microbial, metabolic, and immune-targeted therapies may yield the most durable outcomes [76].

**Figure 3:**
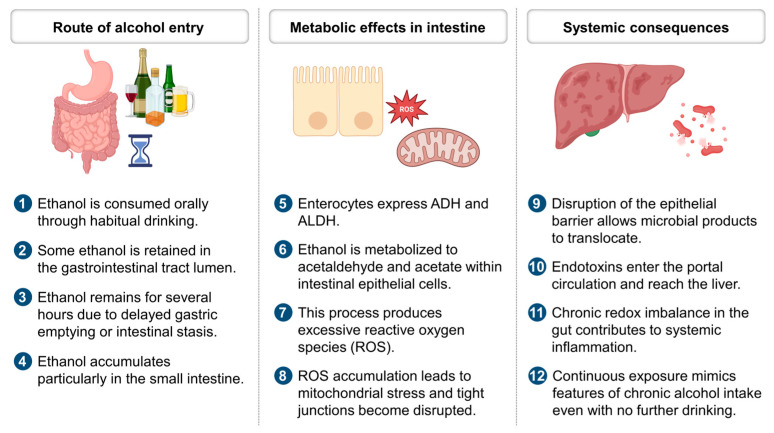
Intestinal ethanol absorption and systemic redox consequences. Ethanol retained in the intestine generates reactive oxygen species and impairs barrier integrity, allowing microbial products to reach the liver and drive systemic redox imbalance. ADH, alcohol dehydrogenase; ALDH, aldehyde dehydrogenase; ROS, reactive oxygen species.

Understanding the immunometabolic consequences of intestinal ethanol stress is essential for developing next-generation interventions. Targeting both innate and adaptive immune axes, alongside epithelial repair and microbial modulation, represents a rational path forward. As illustrated in Figure 3, these interactions form a dynamic and self-perpetuating network that extends from the gut mucosa to systemic compartments, with far-reaching implications for chronic alcohol-associated disease.

## 6. Microbiome–Mitochondria Crosstalk and Translational Interventions

The interplay between gut microbiota and host mitochondria represents a crucial axis in the regulation of redox homeostasis, immune signaling, and metabolic adaptation in alcohol-associated disorders. Ethanol-induced dysbiosis alters the composition and function of the intestinal microbiota, reducing the abundance of beneficial anaerobes, such as *Akkermansia muciniphila* and *Clostridium butyricum*, while promoting the expansion of pathobionts that generate pro-inflammatory and pro-oxidant metabolites [77]. This microbial shift not only impairs epithelial signaling but also compromises mitochondrial function in enterocytes by increasing oxidative load and disrupting metabolic crosstalk.

Emerging evidence suggests that specific microbial taxa and their metabolites regulate mitochondrial biogenesis and redox buffering capacity. Short-chain fatty acids (SCFAs), particularly butyrate, act as histone deacetylase inhibitors and stimulate PGC-1α expression, thereby promoting mitochondrial oxidative phosphorylation and reducing oxidative stress [78]. Ethanol-induced depletion of SCFA-producing microbes contributes to mitochondrial fragmentation, ATP depletion, and oxidative injury in the intestinal epithelium. Additionally, loss of SCFAs impairs the regulation of epithelial autophagy and mitophagy, further diminishing barrier resilience.

Restoring microbial homeostasis through probiotic and postbiotic therapies has shown promise in reversing ethanol-induced mitochondrial dysfunction. Supplementation with *Akkermansia muciniphila* has been shown to improve mucosal integrity, enhance tight junction expression, and reduce lipid peroxidation in ethanol-fed animal models [79]. Co-administration of microbial metabolites, such as indole-3-propionic acid, inosine, and secondary bile acids, appears to activate mitochondrial antioxidant responses through Nrf2 and SIRT1 signaling pathways. These bioactive molecules also promote mitochondrial biogenesis and improve respiratory efficiency.

In parallel, host mitochondrial pathways shape the microbial niche through mechanisms involving NAD^+^ availability, reactive oxygen species, and antimicrobial peptide production. Ethanol-driven inhibition of NAD^+^ biosynthesis disrupts sirtuin activity and impairs mitochondrial protein acetylation homeostasis, leading to redox collapse and altered innate immune surveillance. This disruption facilitates overgrowth of LPS-rich taxa and weakens mucosal tolerance. Emerging studies suggest that NAD^+^ restoration strategies may indirectly remodel microbiota by altering host-derived metabolic signals and epithelial redox status [80].

One innovative therapeutic strategy is fecal microbiota transplantation (FMT), which reintroduces a diverse and functionally competent microbial community into the ethanol-compromised gut. Early-stage clinical trials suggest that FMT may restore microbial and mitochondrial balance, reduce systemic endotoxemia, and improve metabolic markers in patients with alcohol-related liver disease [81]. However, standardization of donor selection, engraftment protocols, and long-term efficacy studies are still needed. Additionally, synthetic microbial consortia and next-generation probiotics may offer controlled alternatives to traditional FMT.

Ultimately, therapeutic modulation of microbiome–mitochondria crosstalk represents a promising frontier in the prevention and management of alcohol-associated redox pathophysiology. As the molecular underpinnings of this bidirectional relationship become clearer, integrative treatment strategies that bridge microbial ecology and host metabolism may offer durable solutions to ethanol-induced systemic injury (Figure 3). Embracing this systems-level perspective could guide the development of multifaceted therapeutics tailored to both microbial and host metabolic networks.

## 7. Strategic Implications and Future Directions

Despite the growing recognition of the small intestine as a critical player in alcohol-associated oxidative stress, its clinical assessment and therapeutic targeting remain underdeveloped. A key challenge lies in the integration of intestinal redox pathology into current diagnostic frameworks, which overwhelmingly rely on hepatic biomarkers and systemic inflammation indices. Emerging data suggest that biomarkers reflecting gut permeability, microbial dysbiosis, and mitochondrial dysfunction, such as serum zonulin, LPS-binding protein, and circulating mtDNA, could enhance diagnostic precision and prognostication in alcohol-associated disease [82].

From a therapeutic standpoint, translating mechanistic insights into effective interventions requires overcoming several barriers. These include inter-individual variability in gut microbiome composition, incomplete understanding of host–microbe–mitochondria interactions, and regulatory hurdles surrounding live biotherapeutics, such as fecal microbiota transplantation. Additionally, while NAD^+^ precursors, FXR agonists, and SCFA-based therapies are promising, their long-term efficacy, bioavailability, and safety profiles in human cohorts remain to be validated through well-powered clinical trials.

Future research should also prioritize the development of integrated therapeutic strategies that combine dietary modulation, microbial interventions, and metabolic correction. Personalized approaches grounded in microbiome and metabolome profiling may offer the greatest therapeutic leverage. Systems biology tools, such as multi-omic network modeling and organoid platforms, will be essential in elucidating the bidirectional feedback loops between the intestine and liver.

Finally, educational and clinical awareness campaigns are necessary to shift the paradigm from a liver-centric to a gut–liver systems model in alcohol-associated disease. This reframing has the potential to identify novel intervention points, enhance early diagnosis, and guide the design of next-generation therapeutics.

## 8. Conclusions

Alcohol-associated oxidative stress is often framed as a hepatic problem, yet this review argues for a paradigm that centers the small intestine as a primary origin of redox imbalance through a mechanism of prolonged intestinal ethanol absorption. The prolonged phase of ethanol absorption perpetuates oxidative stress and immune dysregulation originating in the gut, with subsequent reverberations throughout the liver and beyond. Integrating findings across microbiome shifts, mitochondrial dysfunction, and barrier breakdown, we advocate for early, gut-targeted strategies to disrupt this self-propagating loop. Shifting the focus upstream clarifies the mechanisms involved and identifies actionable points for intervention.

## Abbreviations

The following abbreviations are used in this manuscript:

ADH	Alcohol Dehydrogenase
ALD	Alcohol-Associated Liver Disease
ALDH	Aldehyde Dehydrogenase
AP-1	Activator Protein 1
BAC	Blood Alcohol Concentration
CYP2E1	Cytochrome P450 2E1
FXR	Farnesoid X Receptor
GI	Gastrointestinal
IgA	Immunoglobulin A
IL-6	Interleukin 6
IL-10	Interleukin 10
LPS	Lipopolysaccharide
MEOS	Microsomal Ethanol Oxidizing System
NAD^+^/NADH	Nicotinamide Adenine Dinucleotide (oxidized/reduced)
NF-κB	Nuclear Factor kappa-Light-Chain-Enhancer of Activated B Cells
NMN	Nicotinamide Mononucleotide
NR	Nicotinamide Riboside
Nrf2	Nuclear Factor Erythroid 2–Related Factor 2
PGC-1α	Peroxisome Proliferator-Activated Receptor Gamma Coactivator 1-Alpha
ROS	Reactive Oxygen Species
SCFA	Short-Chain Fatty Acid
SIRT	Sirtuin
TGR5	Takeda G-Protein Receptor 5
TLR4	Toll-Like Receptor 4
TNF-α	Tumor Necrosis Factor Alpha
ZO-1	Zonula Occludens 1
